# Characteristics of *CBL*-mutated patients with chronic myelomonocytic leukemia in a national (ABCMML) and an international cohort (cBIOPORTAL)

**DOI:** 10.1007/s10354-025-01093-9

**Published:** 2025-06-26

**Authors:** Alexandra Plötzeneder, Klaus Geissler

**Affiliations:** Medical School, Sigmund Freud University, Sigmund Freud Platz 3, 1020 Vienna, Austria

**Keywords:** Chronic myelomonocytic leukemia, *CBL*, Austrian biodatabase for chronic myelomonocytic leukemia, cBIOPORTAL, Mutations, Chronische myelomonozytäre Leukämie, *CBL*, Austrian Biodatabase for chronic myelomonocytic leukemia, BioPortal, Mutationen

## Abstract

The big data derived from major international collaborations now makes it possible to validate findings from traditional national patient cohorts in order to demonstrate consistency. We compared findings from *CBL*-mutated patients in the Austrian biodatabase for chronic myelomonocytic leukemia (ABCMML) with those in the CMML cohort recorded in cBIOPORTAL. *CBL* mutations were linked to shorter survival in the ABCMML cohort but not in the cBioPortal cohort. In neither cohort was there an association between AML-free survival and *CBL* mutations. Elevated leukocyte levels were observed in patients with *CBL* mutations in both cohorts. In neither cohort did hemoglobin values, platelet values, and circulating blast levels differ between patients with *CBL* mutations and those without. Data validation using two independent CMML cohorts ensures the quality standards for clinical decision-making.

**Supplementary Information:**

The online version of this article (10.1007/s10354-025-01093-9) contains supplementary material, which is available to authorized users.

## Abstract (de)

Die Validierung nationaler Patientenkohorten zur Überprüfung der Datenkonsistenz wird durch umfangreiche Daten aus internationalen Kooperationen ermöglicht. Wir verglichen die Ergebnisse von Patienten mit *CBL*-Mutation aus der Studie zur nationalen „Austrian Biodatabase for chronic myelomonocytic leukemia“ (ABCMML) mit denen von Patienten, die in der internationalen Plattform BioPortal dokumentiert sind. Bei Patienten mit einer *CBL*-Mutation war die Überlebensdauer in der ABCMML-Kohorte kürzer, jedoch nicht in der BioPortal-Kohorte. In keiner der beiden Kohorten war das akute myeloische Leukämie (AML) freie Überleben mit einer *CBL*-Mutation assoziiert. Die Patienten mit *CBL*-Mutation wiesen in beiden Kohorten höhere Leukozytenwerte auf als die Wildtyp-Patienten. Die Hämoglobinwerte, Thrombozytenzahlen und zirkulierenden Blasten wiesen in beiden Kohorten keinen Unterschied zwischen Patienten mit und ohne *CBL*-Mutation auf. Diese Validierung von Daten in 2 unabhängigen Kohorten mit chronischer myelomonozytärer Leukämie (CMML) gewährleisten die erforderlichen Qualitätsstandards für klinische Entscheidungsfindungen.

## Introduction

Chronic myelomonocytic leukemia (CMML) is a rare hematologic disease in elderly people with a variety of phenotypes and genetic variations that can progress to secondary acute myeloid leukemia (AML). In accordance with the FAB criteria, CMML was first divided into two groups based on myeloproliferation: myelodysplastic syndrome (MD-CMML; WBC count < 13 × G/L) and myeloproliferative disease (MP-CMML; WBC count > 13 × G/L) [1, 2]. Because CMML exhibits traits of both MDS and MPN, the World Health Organization (WHO) categorized it as being in the hybrid category (MDS/MPN) in 2002 [3]. Two groups recently reported updated diagnostic criteria for CMML following the 2016 revision to the WHO’s classification of myeloid neoplasms and acute leukemia [4–6]. The outcomes of CMML patients can vary greatly, indicating that a number of factors influence how the disease progresses and what causes these patients to die [7–13].

We recently reported the Austrian biodatabase for CMML (ABCMML). Patients with CMML have had their epidemiologic, hematologic, biochemical, clinical, immunophenotypic, cytogenetic, molecular, and biologic data gathered from various Austrian centers for 40 years [14]. It has been demonstrated to be a representative and practical source of real-world data for biomedical research.

Because of the molecular heterogeneity of CMML, it is critical to understand the meaning of molecular characteristics so that the patient can be provided with the best possible care for their unique circumstances. The effects of molecular aberrations on the clinical outcome and phenotype of disease have been examined in a few studies, but the majority of these studies’ conclusions were not confirmed by separate cohorts. However, until a prognostic parameter’s usefulness has been established, it should not be used in clinical settings. Evaluation of a prognostic parameter’s performance in a sample different from the one used to build the model is known as external validation [15].

Big data containing a huge number of datasets from international large consortium efforts are now available for many cancer entities, including CMML. The cBioPortal platform is such a collection of big data that aims to build a platform to support clinical decisions for personalized cancer treatment [16]. Moreover, due to the large number of well-characterized patients, it is a perfect source of data for validation of findings in traditional, sometimes much smaller patient cohorts. In this study we used data from CMML patients documented in cBioPortal to validate the features of *CBL*-mutated CMML patients who have been analyzed in the ABCMML.

## Patients and methods

### Patients

#### ABCMML analysis

The ABCMML can serve as a representative and practical real-world data source for biological research, as we have demonstrated recently [13]. Epidemiologic, hematologic, biochemical, clinical, immunophenotypic, cytogenetic, molecular, and biologic data of CMML patients from various sites are gathered retrospectively and included in this database. Leukemic transformation and CMML were diagnosed based on WHO criteria [2–4]. Patient records were used to gather laboratory and clinical information. Prior to analyzing data from institutions, a thorough central manual retrospective chart review was conducted to guarantee data quality. This study did not include CMML patients who were undergoing transition. In 327 patients, mutation data were available to analyze overall survival (OS), acute myeloid leukemia (AML)-free survival, and differences in phenotypic parameters between mutated and wildtype patients. This research was approved by the ethics committee of the City of Vienna on 10 June 2015 (ethic code: 15-059-VK).

#### cBioPortal analysis

We selected the myelodysplastic syndromes dataset from cBioPortal [16] containing 399 CMML cases with data including age, sex, white blood cells (WBC), hemoglobin (Hb), platelets, OS, AML-free survival, bone marrow (BM) blasts, circulating blasts, cytogenetics, and gene mutations (http://www.cbioportal.org) to analyze OS, AML-free survival, and differences in phenotypic parameters between mutated and nonmutated patients.

### Statistical analysis

To investigate whether particular factors were connected to OS, the log-rank test was employed. Overall survival was defined as either until the final follow-up (censored) or as the period from sampling to death (uncensored). The duration between sampling and either transformation into AML or death (uncensored) or last follow-up (censored) was referred to as the AML-free survival. The chi-squared test was used to compare dichotomous variables between groups. When continuous variables were not normally distributed, two unmatched groups were compared using the Mann–Whitney U test. Results were considered significant at *p* < 0.05. Statistical analyses were performed with SPSS v. 27 (IBM Corp., Armonk, NY, USA); the reported *p*-values were two sided. In the ABCMML database, mutations with a variant allele frequency (VAF) of at least 5% are considered positive and in the cBioPortal platform with a VAF of at least 2%.

## Results

### Characteristics of patients and prevalences of *CBL* mutations

The baseline characteristics of both CMML cohorts are shown in supplementary Tables 1 and 2. In the ABCMML cohort 327 patients were analyzed and in the cBioPortal cohort 399 patients. As seen in other CMML series, there was a male predominance among CMML patients in both cohorts, and more than half of patients were aged 70 years or older [13]. All characteristics except leukocytes were comparable between cohorts. The proportion of patients with leukocytes > 13 G/L was significantly higher in the ABCMML cohort compared to the cBioPortal cohort (57% vs. 32%, *p* < 0.001). The median leukocyte counts were 14.1 vs. 9.2 G/L in these cohorts, respectively. Regarding clinical outcome, the median survival was 29.0 months in the ABCMML cohort as compared to 31.6 months in the cBioPortal cohort. The prevalences of *CBL* mutations were 15.2% (50/327) in the ABCMML group and 10.2% (41/399) in the BIOPOPRTAL group.

### Impact of *CBL *mutations on survival and AML-free survival

**Fig. 1:**
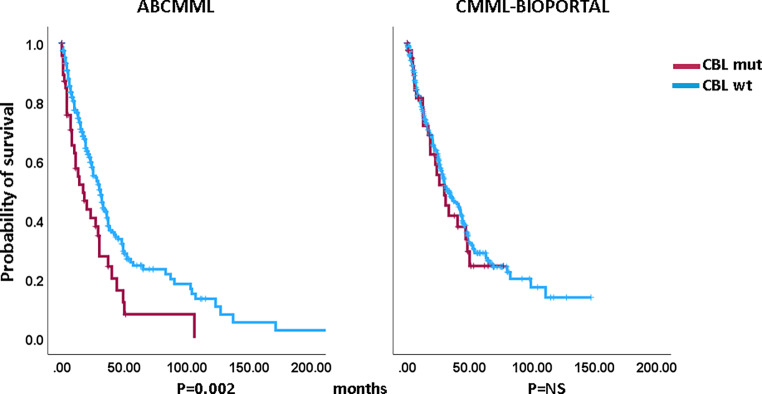
Kaplan–Meier plots for overall survival in CMML patients with and without *CBL* mutations

**Fig. 2:**
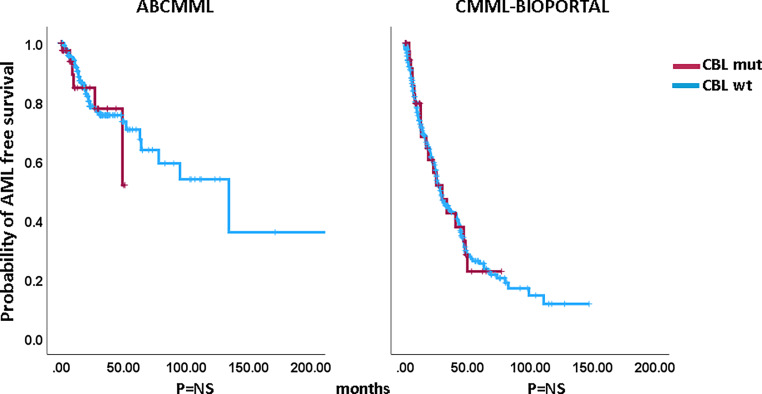
Kaplan–Meier plots for AML-free survival in CMML patients with and without *CBL* mutations

Figures 1 and 2 show the Kaplan–Meier curves of OS in *CBL*-mutated (variants and variant allele frequencies are shown in supplementary Tables 3 and 4) and *CBL*-nonmutated patients in both cohorts. Whereas in the ABCMML cohort the median survival of the *CBL*-mutated patients was significantly shorter than in *CBL*-nonmutated patients, there was no difference in survival in the cBioPortal cohort. The median survival of *CBL*-mutated patients was 17.0 vs. 31.0 months (*p* = 0.002) in the ABCMML patients and 29.7 vs. 31.7 months (*p* = 0.625) in the cBioPortal patients. Regarding AML-free survival, there was no significant difference between *CBL*-mutated and *CBL*-nonmutated patients in either cohort. The median AML-free survival was not reached vs. 134.0 months (*p* = 0.608) in the ABCMML cohort and 29.7 vs. 28.1 months (*p* = 0.976) in the cBioPortal cohort.

### Laboratory features in the presence or absence of *CBL* mutations

**Fig. 3:**
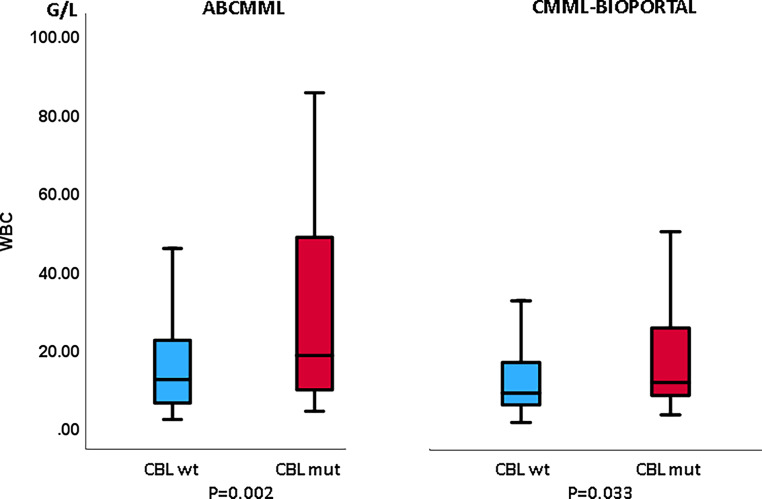
Boxplots showing the distribution of leukocytes in *CBL*-nonmutated and *CBL*-mutated CMML patients including median values, minimum values, maximum values, and upper and lower quartiles in both study cohorts

**Fig. 4:**
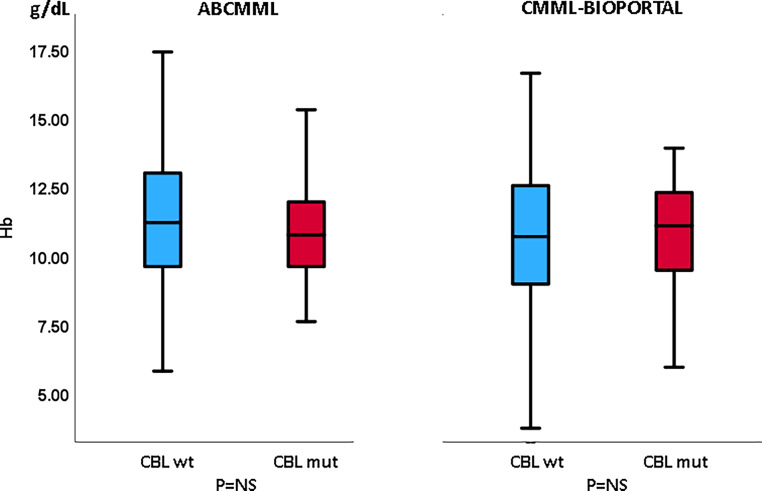
Boxplots showing the distribution of hemoglobin values in *CBL*-nonmutated and *CBL*-mutated CMML patients including median values, minimum values, maximum values, and upper and lower quartiles in both study cohorts

**Fig. 5:**
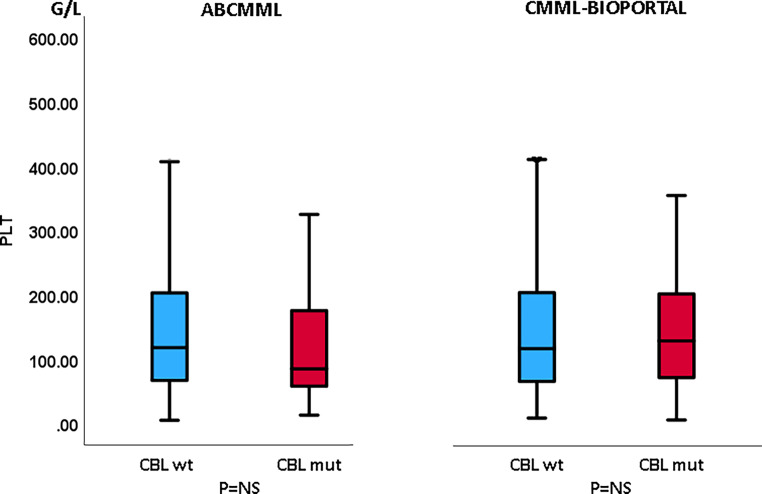
Boxplots showing the distribution of platelets in *CBL*-nonmutated and *CBL*-mutated CMML patients including median values, minimum values, maximum values, and upper and lower quartiles in both study cohorts

Tables 1 and 2 show the phenotypic parameters in the ABCMML and the cBioPortal patients, respectively. There was a significantly higher proportion of patients with circulating blasts in the ABCMML cohort, whereas this was not the case in the cBioPortal cohort. Regarding the proportion of patients with leukocytosis > 13 G/L, anemia with Hb < 10 g/dl, and thrombocytopenia with platelet values < 100 G/L, there was no significant difference between *CBL*-mutated and nonmutated patients in either cohort. In Figs. 3, 4, and 5, metric values are visualized by boxplot diagrams. In the ABCMML cohort, the median values of *CBL*-mutated and nonmutated patients were 18.3 vs. 12.2 G/L for WBC, 11.2 vs. 10.8 g/dL for Hb, and 85 vs. 118 G/L for platelets, respectively. In the cBioPortal cohort, the median values of *CBL*-mutated and nonmutated patients were 11.7 vs. 9.0 G/L for WBC, 11.1 vs. 10.7 g/dL for Hb, and 130 vs. 118 G/L for platelets, respectively.

**Table 1:** Phenotypic features of ABCMML patients including leukocytosis, anemia, thrombocytopenia, and circulating blasts stratified by the presence or absence of a *CBL* mutation

Parameter	With *CBL *mutation	Without *CBL *mutation	*P*-value
WBC ≥ 13 G/L	29/48 (60%)	123/268 (46%)	0.084
Hb < 10 g/dL	14/48 (29%)	86/268 (32%)	0.739
PLT < 100 G/L	27/48 (56%)	109/269 (41%)	0.057
PB blasts present	16/44 (36%)	45/221 (20%)	0.030

**Table 2:** Phenotypic features of cBioPortal patients including leukocytosis, anemia, thrombocytopenia, and circulating blasts stratified by the presence or absence of a *CBL* mutation

Parameter	With *CBL *mutation	Without *CBL *mutation	*P*-value
WBC ≥ 13 G/L	15/38 (39%)	106/345 (31%)	0.275
Hb < 10 g/dL	12/41 (29%)	135/356 (38%)	0.310
PLT < 100 G/L	16/41 (39%)	139/351 (40%)	1.000
PB blasts present	12/32 (38%)	76/301 (25%)	0.143

## Discussion

In this study we analyzed a national CMML cohort from Austria (ABCMML) and an international cohort of CMML patients (cBioPortal) regarding clinical, epidemiologic, and hematologic features of *CBL*-mutated patients in order to get information on the consistency and general validity of findings.

A major finding of this study is the fact that in the ABCMML cohort, patients with *CBL* mutations had significantly shorter survival than wildtype patients, but in the cBioPortal cohort there was no difference. Looking at published series, *CBL* was an adverse risk factor in a CMML cohort published by Itzykson in 2013 [11] and also in an international dataset published by Padron in 2015 [17]. *CBL*, however, was not an adverse risk factor in an Italian–Spanish cohort reported in 2016 by Elena [12], nor in a Chinese cohort reported by Nie in 2022 [13]. Due to the fact that patients had been included in the ABCMML database since 1980, whereas the CMML patients captured in the cBioPortal cohort were included in recent years, it seems that the prognostic impact of the *CBL* mutation becomes lost in later series. Since one can assume that the proportion of patients with no access to hypomethylating agent (HMA) treatment was higher in earlier CMML series, one could speculate that HMA may overcome the negative impact of this mutation on survival. Of course, this speculation needs to be analyzed in future studies.

The correlation of phenotypic features with mutational status in CMML patients has been described previously [18]. Molecular aberrancies in the RAS/MAPK signaling pathway have been shown to be associated with leukocytosis, myeloproliferation, and increased blast cells [19–21]. For both cohorts in our study, it is consistently shown that *CBL* mutations are associated with an increased white blood cell count. This observation is in line with the fact that *CBL* is considered to be a component using the RAS signaling pathway. There was no impact of the *CBL* mutation on other phenotypic parameters such as hemoglobin and platelet values.

Another finding in this study was the fact that the proportion of patients with leukocytes > 13 G/L was significantly higher in the ABCMML cohort as compared to the cBioPortal cohort. The reason for this imbalance is not completely clear. Increased laboratory screening in recent years among asymptomatic persons may detect some diseases, including CMML, in an earlier phase than in the past. Therefore, older patient series may be enriched in patients with more advanced disease as compared to more recent series. In fact, we have seen a significant drop in the proportion of patients with MP-CMML from 66% to 48% since 2010 in the ABCMML database (unpublished data).

Limitations to this study are the retrospective nature of the data collection with missing data and the fact that in the ABCMML cohort, changes to the diagnostic criteria of CMML have occurred over time since its first description in 1982, suggesting that the ABCMML database is more heterogenous compared to the cBioPortal group, which contains patients who were included over a shorter period of time. Furthermore, it needs to be considered that a proportion of patients in ABCMML, in particular older patients, did not consent to BM puncture. However, we do not think that this greatly affected diagnostic accuracy, since persistent peripheral blood monocytosis is the most important diagnostic feature, and a genoclinical model has been recently described that uses mutational data, peripheral blood values, and clinical variables to predict the MDS vs. CMML diagnosis with high accuracy in the absence of a BM biopsy result [22]. Moreover, somatic mutations associated with CMML were not only detected in CMML patients confirmed by BM biopsy but also in 57% of patients with nondiagnostic BM features. Interestingly, the OS in mutated patients without BM diagnosis of CMML was indistinguishable from mutated patients with this BM diagnosis, suggesting that that the mutational spectrum is a much more sensitive parameter for detection of myeloid malignancies than BM morphology [23].

Recently, the disease-centered approach to health care administration has given way to a patient-centered approach [24]. The implementation of precise and personalized medicine based on individualized information will be made possible by the adoption of big data, which is defined by a vast volume of digital data that is continuously generated by people in clinical treatment and daily life. Furthermore, as we have demonstrated in this work, these data can be utilized to validate results from national cohorts, which helps to improve patient care.

## Supplementary Information

**Supplementary Table 1:** Patient characteristics in the ABCMML cohort

**Supplementary Table 2:** Patient characteristics in the CMML cBioPortal cohort

**Supplementary Table 3:** *CBL* variants and variant allele frequencies in patients of the ABCMML and genetic variants with unknown protein consequence

**Supplementary Table 4:** *CBL* variants and variant allele frequencies in patients of the cBioPortal

## References

[1] Bennett JM, Catovsky D, Daniel MT, et al. Proposals for the classification of the myelodysplastic syndromes. Br J Haematol. 1982;51(2):189–99. 6952920

[2] Vardiman JW, Harris NL, Brunning RD. The world health organization (WHO) classification of the myeloid neoplasms. Blood. 2002;100(7):2292–302. 12239137 10.1182/blood-2002-04-1199

[3] Vardiman JW, Thiele J, Arber DA, et al. The 2008 revision of the world health organization (WHO) classification of myeloid neoplasms and acute leukemia: rationale and important changes. Blood. 2009;114(5):937–51. 19357394 10.1182/blood-2009-03-209262

[4] Arber DA, Orazi A, Hasserjian R, et al. The 2016 revision to the world health organization classification of myeloid neoplasms and acute leukemia. Blood. 2016;127(20):2391–405. 27069254 10.1182/blood-2016-03-643544

[5] Arber DA, Orazi A, Hasserjian RP, et al. International consensus classification of myeloid neoplasms and acute leukemias: integrating morphologic, clinical, and genomic data. Blood. 2022;140(11):1200–28. 35767897 10.1182/blood.2022015850 PMC9479031

[6] Khoury JD, Solary E, Abla O, et al. The 5th edition of the world health organization classification of haematolymphoid tumours: myeloid and histiocytic/dendritic neoplasms. Leukemia. 2022;36(7):1703–19. 35732831 10.1038/s41375-022-01613-1 PMC9252913

[7] Patnaik MM, Tefferi A. Chronic myelomonocytic leukemia: 2020 update on diagnosis, risk stratification and management. Am J Hematol. 2020;95(1):97–115. 31736132 10.1002/ajh.25684

[8] Onida F, Kantarjian HM, Smith TL, et al. Prognostic factors and scoring systems in chronic myelomonocytic leukemia: a retrospective analysis of 213 patients. Blood. 2002;99(3):840–9. 11806985 10.1182/blood.v99.3.840

[9] Jankowska AM, Makishima H, Tiu RV, et al. Mutational spectrum analysis of chronic myelomonocytic leukemia includes genes associated with epigenetic regulation: UTX, EZH2, and DNMT3A. Blood. 2011;118(14):3932–41. 21828135 10.1182/blood-2010-10-311019 PMC3193268

[10] Kohlmann A, Grossmann V, Klein HU, et al. Next-generation sequencing technology reveals a characteristic pattern of molecular mutations in 72.8 % of chronic myelomonocytic leukemia by detecting frequent alterations in TET2, CBL, RAS, and RUNX1. J Clin Oncol. 2010;28(24):3858–65. 20644105 10.1200/JCO.2009.27.1361

[11] Itzykson R, Kosmider O, Renneville A, et al. Prognostic score including gene mutations in chronic myelomonocytic leukemia. J Clin Oncol. 2013;31(19):2428–36. 23690417 10.1200/JCO.2012.47.3314

[12] Elena C, Gallì A, Such E, et al. Integrating clinical features and genetic lesions in the risk assessment of patients with chronic myelomonocytic leukemia. Blood. 2016;128(10):1408–17. 27385790 10.1182/blood-2016-05-714030 PMC5036538

[13] Nie Y, Shao L, Zhang H, He CK, Li H, Zou J, et al. Mutational landscape of chronic myelomonocytic leukemia in chinese patients. Exp Hematol Oncol. 2022;11(1):32. 35610628 10.1186/s40164-022-00284-z PMC9128105

[14] Geissler K, Jäger E, Barna A, et al. The austrian biodatabase for chronic myelomonocytic leukemia (ABCMML): a representative and useful real-life data source for further biomedical research. Wien Klin Wochenschr. 2019;131(17-18):410–8. 31321531 10.1007/s00508-019-1526-1 PMC6748886

[15] Royston P, Altman DG. External validation of a cox prognostic model: principles and methods. BMC Med Res Methodol. 2013;13(1):33. 23496923 10.1186/1471-2288-13-33 PMC3667097

[16] Cerami E, Gao J, Dogrusoz U, et al. The cbio cancer genomics portal: an open platform for exploring multidimensional cancer genomics data. Cancer Discov. 2012;2(5):401–4. 22588877 10.1158/2159-8290.CD-12-0095 PMC3956037

[17] Padron E, Garcia-Manero G, Patnaik MM, et al. An international data set for CMML validates prognostic scoring systems and demonstrates a need for novel prognostication strategies. Blood Cancer Journal. 2015;31;5(7):e333. 10.1038/bcj.2015.53 PMC4526779 26230957

[18] Itzykson R, Solary E. An evolutionary perspective on chronic myelomonocytic leukemia. Leukemia. 2013;27(7):1441–50. 23558522 10.1038/leu.2013.100

[19] Ricci C, Fermo E, Corti S, et al. RAS Mutations Contribute to Evolution of Chronic Myelomonocytic Leukemia to the Proliferative Variant. Clin Cancer Res. 2010;15;16(8):2246–56. 10.1158/1078-0432.CCR-09-2112 20371679

[20] Cervera N, Itzykson R, Coppin E, et al. Gene mutations differently impact the prognosis of the myelodysplastic and myeloproliferative classes of chronic myelomonocytic leukemia. Am J Hematol. 2014;89(6):604–9. 24595958 10.1002/ajh.23702

[21] Schuler E, Schroeder M, Neukirchen J, et al. Refined medullary blast and white blood cell count based classification of chronic myelomonocytic leukemias. Leuk Res. 2014;38(12):1413–9. 25444076 10.1016/j.leukres.2014.09.003

[22] Radakovich N, Meggendorfer M, Malcovati L, et al. A geno-clinical decision model for the diagnosis of myelodysplastic syndromes. Blood Adv. 2021;5(21):4361–9. 34592765 10.1182/bloodadvances.2021004755 PMC8579270

[23] Cargo C, Cullen M, Taylor J, et al. The use of targeted sequencing and flow cytometry to identify patients with a clinically significant monocytosis. Blood. 2019;133(12):1325–34. 30606702 10.1182/blood-2018-08-867333

[24] Batko K, Ślęzak A. The use of big data analytics in healthcare. J Big Data. 2022;9(1):3. 35013701 10.1186/s40537-021-00553-4 PMC8733917

