# Supplementary material for: Characteristics of CBL-mutated patients with chronic myelomonocytic leukemia in a national (ABCMML) and an international cohort (cBIOPORTAL)
Source: Wien Med Wochenschr. 2025 Jun 26;175(11-12):282–8. doi: 10.1007/s10354-025-01093-9 (PMC12380883; doi:10.1007/s10354-025-01093-9)
Supplement: Supplementary file 3 — Supplementary Table 3: CBL variants and variant allele frequencies in patients of the ABCMML and genetic variants with unknown protein consequence [file 10354_2025_1093_MOESM3_ESM.docx]

**Suppl Table 3:** *CBL* variants and variant allele frequencies in patients of the ABCMML

| **ABCMML ID** | **CBL** | **VAF** |
| --- | --- | --- |
| CMML_1_007 | C381R | 52 |
| CMML_1_103 | C416W | 100 |
| CMML_1_107 | T371H | 17 |
| CMML_1_115 | Q366fs | 39 |
| CMML_1_122 | L380P | 43 |
| CMML_1_125 | C404W | 88 |
| CMML_1_126 | K382Q | 69 |
| CMML_1_143 | S376P | 5 |
| CMML_1_146 | C416Y | 56 |
| CMML_3_176 | C381W | 34 |
| CMML_3_178 | R420G | 91 |
| CMML_14_301 | C416S | 6 |
| CMML_14_316 | &c1096-7A>G | 41 |
| CMML_17_478 | H398N | 21 |
| CMML_16_363 | C416S | 12 |
| CMML_16_365 | A420Q | 12 |
| CMML_18_435 | G413D | 97 |
| CMML_18_437 | P417S | 92 |
| CMML_1_464 | N390H | 51 |
| CMML_1_467 | C381Y | 31 |
| CMML_1_472 | H398Y | 6 |
| CMML_18_524 | C418S | 76 |
| CMML_1_527 | R420L | 79 |
| CMML_1_529 | R420L | 38 |
| CMML_18_536 | F418S | 87 |
| CMML_1_543 | C404R | 33 |
| CMML_1_571 | D390V | 5 |
| CMML_1_572 | Y371H | 72 |
| CMML_1_574 | D460del | 6 |
| CMML_1_576 | F418S | 39 |
| CMML_1_583 | P417R | 30 |
| CMML_1_595 | C404Y | 10 |
| CMML_1_637 | L380P | 50 |
| CMML_1_659 | Y368_E369del | 82 |
| CMML_18_671 | C384Y | 39 |
| CMML_1_688 | C381Y | 24 |
| CMML_1_691 | R420Q | 19 |
| CMML_1_707 | W509* | 21 |
| CMML_1_712 | &c1228-2A>g | 5 |
| CMML_1_718 | C396Y | 95 |
| CMML_3_177 | C419Y | 85 |
| CMML_14_296 | C404Y | 42 |
| CMML_2_326 | C384Y | 62 |
| CMML_16_329 | Y371H | 28 |
| CMML_16_332 | C381S | 56 |
| CMML_18_429 | Y371H | 40 |
| CMML_1_463 | L380P | 19 |
| CMML_4_619 | P417S | 16 |
| CMML_1_648 | Y371C | 44 |
| CMML_1_654 | C384R | 7 |

& genetic variants with unknown protein consequence
