# Supplementary material for: Characteristics of CBL-mutated patients with chronic myelomonocytic leukemia in a national (ABCMML) and an international cohort (cBIOPORTAL)
Source: Wien Med Wochenschr. 2025 Jun 26;175(11-12):282–8. doi: 10.1007/s10354-025-01093-9 (PMC12380883; doi:10.1007/s10354-025-01093-9)
Supplement: Supplementary file 4 — Supplementary Table 4: CBL variants and variant allele frequencies in patients of the cBioPortal [file 10354_2025_1093_MOESM4_ESM.docx]

**Suppl Table 4:** *CBL* variants and variant allele frequencies in patients of the BIOPORTAL

| **BIOPORTAL ID** | **CBL** | **VAF** |
| --- | --- | --- |
| E-H-100121 | R420Q | 3 |
| E-H-100160 | Q367R | 8 |
| E-H-100207 | Y371H | 49 |
| E-H-100214 | P420Q | 19 |
| E-H-100220 | R420G | 3 |
| E-H-100305 | R420Q | 14 |
| E-H-100307 | R420Q | 57 |
| E-H-100331 | X290_splice | 29 |
| E-H-100361 | Y371H | 3 |
| E-H-103050 | C384S | 47 |
| E-H-103057 | R420Q | 74 |
| E-H-103070 | W303* | 4 |
| E-H-103092 | R420Q | 22 |
| E-H-105461 | X409_splice | 26 |
| E-H-105492 | P417S | 57 |
| E-H-105652 | L399R | 44 |
| E-H-105658 | R420Q | 2 |
| E-H-105945 | R540* | 4 |
| E-H-105970 | T273Rfs*5 | 43 |
| E-H-106240 | C384R | 2 |
| E-H-110402 | R420Q | 11 |
| E-H-110788 | P347Lfs*3 | 2 |
| E-H-110825 | S376F | 42 |
| E-H-110856 | R149* | 16 |
| E-H-116481 | Y371S | 4 |
| E-H-116499 | R462* | 2 |
| E-H-116587 | C381R | 48 |
| E-H-116589 | H398Y | 40 |
| E-H-116710 | D390Y | 6 |
| E-H-116768 | C381Y | 40 |
| E-H-117969 | K382E | 16 |
| E-H-118221 | Q367P | 70 |
| E-H-118444 | X290_splice | 14 |
| E-H-118493 | C384Y | 5 |
| E-H-120855 | C381Y | 19 |
| E-H-120876 | C381Y | 29 |
| E-H-120888 | R420Q | 59 |
| E-H-120991 | L399R | 80 |
| E-H-121139 | Q249* | 33 |
| E-H-121140 | X522_splice | 6 |
| E-H-131861 | R718* | 4 |
